# Does Postmodernism Really Entail a Disregard for the Truth? Similarities and Differences in Postmodern and Critical Rationalist Conceptualizations of Truth, Progress, and Empirical Research Methods

**DOI:** 10.3389/fpsyg.2020.545959

**Published:** 2020-09-17

**Authors:** Peter Holtz

**Affiliations:** IWM Leibniz-Institut für Wissensmedien, Tübingen, Germany

**Keywords:** postmodernism, critical rationalism, epistemology, philosophy of science, Kenneth Gergen, Karl Popper, David Deutsch, Jean François Lyotard

## Abstract

Within this article, I will compare postmodernist and critical rationalist conceptualizations of epistemological key concepts such as truth, progress, and research methods. An analysis of Gergen’s program for a postmodern psychology shows that a naïve positivist understanding of truth is clearly incompatible with his postmodernist approach, whereas a correctly understood falsificationist use of truth as a guiding ideal may not be. However, postmodernists are often content with a diversity of voices as the endpoint of scientific activities, whereas critical rationalists such as Popper would put more emphasis on attempts to reach a common understanding. The differences between critical rationalists such as Popper and Deutsch and postmodernists such as Gergen are more complicated when it comes to conceptualizations of progress: whereas, postmodernists do not deny the existence of some forms of progress such as technological innovation, they argue that the modernist grand narrative, which views Western culture and the corresponding technological revolutions as being equal to epistemological progress and societal and political progress *per se*, has become untenable. Debates on possible negative consequences of modern technology are one example of evidence for this. Here, critical rationalists tend to engage in a legitimization discourse, sensu Lyotard, and to defend Western culture with all its deficiencies as a necessary precondition for evolutionary epistemic as well as societal and political progress, although they would agree with large parts of the postmodern critique of modernism. Postmodernists and critical rationalists would both agree that psychology as a field would benefit greatly, among other things, from a transition from a methods-oriented approach to scientific knowledge to a more problem-oriented approach, and from less methodological dogmatism. Taken together, postmodernism and critical rationalism may not be as irreconcilable as it may seem at first glance.

## Introduction

There are numerous examples of philosophers as well as psychologists and other intellectuals warning of the destructive powers of the obscure specter of postmodernism (cf. Jauß, 1983), which to them is apparently haunting not only modern psychology, but also all of contemporary science and society. The psychologist and internet personality Jordan B. Peterson defines postmodernism on his homepage as follows (Peterson, undated):

Postmodernism is essentially the claim that (1) since there are an innumerable number of ways in which the world can be interpreted and perceived (and those are tightly associated) then (2) no canonical manner of interpretation can be reliably derived.

That’s the fundamental claim. An immediate secondary claim (and this is where the Marxism emerges) is something like “since no canonical manner of interpretation can be reliably derived, all interpretation variants are best interpreted as the struggle for different forms of power.”

In a similar vein, theoretical physicist and critical rationalist philosopher David Deutsch describes postmodernism as follows (Deutsch, 2011, p. 314):

One currently influential philosophical movement goes under various names, such as postmodernism, deconstructionism, and structuralism [sic!], depending on historical details that are unimportant here. It claims that because all ideas, including scientific theories, are conjectural and impossible to justify, they are essentially arbitrary: they are no more than stories, known in this context as ‘narratives’. Mixing extreme cultural relativism with other forms of anti-realism, it regards objective truth and falsity, as well as reality and knowledge of reality, as mere conventional forms of words that stand for an idea’s being endorsed by a designated group of people such as an elite or consensus, or by a fashion or other arbitrary authority. And it regards science and the Enlightenment as no more than one such fashion, and the objective knowledge claimed by science as an arrogant cultural conceit.

Psychologist and linguist David Pinker recently made the following statement in an interview with the British newspaper *The Guardian* (Anthony, 2018):

If scientific beliefs are just a particular culture’s mythology, how come we can cure smallpox and get to the moon, and traditional cultures cannot? And if truth is just socially constructed, would you say that climate change is a myth? It’s the same with moral values. If moral values are nothing but cultural customs, would you agree that our disapproval of slavery or racial discrimination or the oppression of women is just a western fancy?

One difficulty in disentangling all this criticism of some diffuse notion of postmodernism is that those who came to be known as the founding fathers and mothers of postmodernism, such as Foucault, Derrida, Lacan, and Irigaray, had never used the term themselves (Wilterdink, 2002, p. 197). Hence, in order to assess the veracity of the claims by Peterson, Deutsch, Pinker, and others, one must first narrow down the notion of postmodernism that is to be analyzed.

Undoubtedly, Lyotard (1979/1984) has largely contributed to the popularization of the term postmodernism in its current meaning with his book on the *postmodern condition*, which – according to Google Scholar – has, as of now, been cited more than 30,000 times in scientific texts. For the field of psychology, the explicit agenda of Gergen (1990) for a postmodern psychology seems to be a seminal text that can serve as a point of reference for the postmodernist movement in the field of psychology.

Based mainly on Gergen’s conceptualization of postmodernism (see also Gergen, 1994, 2001) and to some degree “classic” of Lyotard (1979/1984), I will begin by discussing implications for the field of psychology, to lead into a discussion of differences and similarities between postmodernist and critical rationalist conceptualizations of truth, progress, and empirical research methods.

## Postmodernism

### Modernism

The term *postmodernism* originated in fields as different as philosophy, architecture, and literary theory in the early twentieth century (Wilterdink, 2002). The common element among different conceptualizations of postmodernism seems to be the idea that another era, *modernism*, had reached its end and was to be replaced by a yet-to-be-named new epoch. On a side note, I would like to add to the existing accounts of the origins of postmodernism that in the early twentieth century, the term *hypermodernism* (Tartakower, 1924) was fiercely debated in the world of chess: previously, a modernist movement mirroring the scientific method in trying to discover abstract rules for identifying promising and detrimental positional features and general strategic patterns had replaced an earlier, more romantic approach to chess that focused mostly on mating attacks (e.g., Tarrasch, 1912). In the early twentieth century, a group of grandmasters such as Reti and Nimzovitch had discovered that there were exceptions to the modernist “laws” of good chess that could be used to skillfully outmaneuver dogmatic modernists. However, no hypermodernist at the time denied that the modernist principles were valid for most chess positions. Rather, the object of criticism was the dogmatic and oversimplifying tone of modernism.

In a similar vein, the postmodern movement in the sciences should also be understood first and foremost as a countermovement against some extreme form of modernism (see also Lyotard, 1979/1984, pp. 11–14). Gergen (1990) describes psychology itself as a through-and-through modernist attempt to replace earlier humanities-based approaches to understanding the human condition with a profoundly empirical approach based on the “scientific method” of the natural sciences (for a discussion of the naive positivistic way in which the scientific method is usually understood in psychology, see Holtz and Monnerjahn, 2017). This “modernist romance” (p. 25) is, according to Gergen, characterized by four overarching presuppositions:

1. Basic subject matter: there is something that can be known – be it behavior and its causes or internal processes, such as thoughts or memories and the like. The important thing is that although we do not (yet) know everything, there is agreement (apart from some “localized conflicts”; Gergen, 1990, p. 25) on the subject matter that is to be known and on the fact that the subject matter can be known.
2. Universal properties: modernists assume that they can derive by inductive reasoning from single observations certain abstract, general, and time-invariant laws of nature that explain a class of phenomena that are related to the basic subject matter.
3. Empirical method: in line with what Gergen calls a “logical empiricist” (p. 26) approach (without providing further references), modernists believe that through strict application of the scientific method they can gain true knowledge about the universal properties of the basic subject matter. They believe they can thus prevent the “entry of ideology, values or passions into the description and explanation of relevant phenomena” (p. 26).
4. Research as progressive: by applying the scientific method, scientists can abandon false beliefs and “move toward the establishment of reliable, value neutral truths about our designated segment of the objective world” (p. 26).

Gergen continues by providing a very rough account of various forms of criticism of the logical empiricist’s (or logical positivist’s) philosophy supposedly underlying psychology’s modernism, represented by philosophers such as Quine, Popper, and Kuhn. It seems pretty clear that Gergen is here and elsewhere not referring to certain philosophers from the logical positivist tradition in particular, but to psychology’s (or more specifically social psychology’s) positivism-influenced epistemological tradition (see also Pettigrew, 1991; Holtz and Monnerjahn, 2017).

In the following paragraph, I will outline how and why the modernist dream of positive knowledge about a segment of the objective world has in my opinion pretty clearly reached its limits in the present time. I will use a technology-focused approach similar to Lyotard (1979/1984) in discussing the internet as a research object.

### The Internet as a Thoroughly Postmodern Phenomenon

If we apply those four presuppositions of modernism to the internet, the modernist will quickly run into some problems: the basic subject matter, for example, the internet and specific internet-related activities, came into being only two and a half decades ago. At least those who remember the internet from its early days will certainly agree that what is there and what can be done now does not have much to do with the humble beginnings in the 1990s. And even worse, I assume that none of us will doubt that “the internet” or whatever it is going to evolve into will, 25 years from now, not have much in common with what we are experiencing now.

Furthermore, the internet already offers so many facets and services that the experiences possible within this environment can hardly be subsumed under the same overarching theoretical principles (see also Orben, 2020). That is, of course, based on the assumption that the internet does not change us and is only shaped by us (or other things such as economic interests) driven by psychological principles that also exist outside and beyond the internet. As Gergen (1973) had argued elsewhere, not only is the idea that the environment does not influence the psyche untenable, it is also highly probable, given that most psychological articles end with a concluding statement emphasizing the societal impact of the research reported, that our psychological theorizing about the world influences the very world we analyze. At the very least, we either must admit that our research is without societal consequences or we must abandon the idea of general time-invariant laws that govern human’s minds and behavior.

In view of the internet’s ever-changing subject matter, it is also untenable to think that experiments or any other empirical research method can guarantee the discovery of true statements and the abandonment of false statements. Much time has passed since vision of Allport (1924) that in the “near future,” psychology will have discovered the basic psychic processes that lead to complex social structures, so that “social objects” (such as groups, societies, norms, cultural codes…) would finally lose any explicatory function. Postmodernists such as Gergen demand that this dream of an explanation of the social based upon processes within individuals be finally put to rest for good. In contrast, human individuality, including processes such as thinking and arguing, as well as behavior itself, can only be understood against the background of a cultural fabric that underlies all psychic processes.

Within the internet, it also becomes obvious how knowledge has become a good that is traded just like other goods and that acquires its worth from supply and demand. In earlier days, the production of new knowledge and the administration of existing knowledge had been by and large the privilege of a dedicated class within society such as first priests and monks and then scientists at universities who were employed by and acted in the interest of the respective religious and secular authorities. Knowledge was in these days more of an “end in itself” (Lyotard, 1979/1984, p. 5) than it is today. Lyotard (1979/1984) goes to great lengths to explain how knowledge has already become (as of the 1980s) and will become even more in the future (i.e., now) *the* “principle force of production” (p. 16) that drives our globalized economy. Hence, all claims to knowledge are necessarily also of economic and political interest. Any claims to “value-neutrality” can be nothing more than a sales argument in a globalized knowledge economy.

Knowledge is and will be produced in order to be sold, it is and will be consumed in order to be valorized in a new production: in both cases, the goal is exchange. Knowledge ceases to be an end in itself, it loses its “use-value” (p. 15/16).

Lastly, there is the question of progress. Several arguments can be made against the modernist idea of continuous progress. On the one hand, changes in the subject matter can make something that at time T1 was progress obsolete at T2. Let us imagine that Kraut et al. (1998) “internet paradox,” the idea that the internet as a communication technology eventually makes users lonelier, was true in the 1990s, but untrue in the 2000s. We can also easily imagine that changes in technology could make the statement true again in the 2010s or 2020s. So, what is progress and how can we know that we have made progress?

The question of progress can also be tackled from a very different perspective: what *is* progress? Does progress mean better control over people’s behavior on the internet? Catering to psychological needs with better technologies? Better health and less loneliness? What if abandoning the internet along with any related research was the best progress achievable, as some technology pessimists seem to imply? And who decides what progress is and on which basis? These are some of the core questions postmodernists ask modernists.

### Gergen’s Vision for a Postmodern Psychology

As we have seen, using the scientific method to identify time and context-invariant psychological laws of nature is not feasible from a postmodern point of view. So, what remains to be done for the postmodern psychologist? Already in his earlier works, Gergen (1978) had introduced the ideal of creating *generative theories*, that is, theories that allow for challenging established assumptions about the world and for exploring alternative lifestyles and behavioral patterns. To Gergen (1990), postmodernism opens up a whole new realm of possibilities: whereas psychologists in the modern age were assigned the finally doomed Sisyphus task of finding natural laws where there may be only historically and culturally shaped transient and volatile patterns, they can now, for example, contribute instead to the creation of a better world. In the modernist vision of science, the scientist is a passive observer and analyst. In the postmodern vision, scientists can use their locally and temporally limited knowledge to explore possible worlds, and they can try to answer the question how a change for the better can be facilitated – from a certain perspective at a certain point in time within a given socio-historical context.

## Critical Rationalism

### In a Nutshell

In the following paragraph, I will attempt to summarize the central “mantra” of critical rationalism as concisely as possible. More elaborate accounts of critical rationalism, and the rather misleading way in which it is most often characterized in psychological texts, have been provided elsewhere (e.g., Holtz, 2016; Holtz and Monnerjahn, 2017; Holtz and Odağ, 2018). Gergen’s criticism is targeting primarily modernist psychology and not the “natural sciences,” hence I will refer as a primary point of reference to essay of Popper (1976/1969) *the logic of the social sciences* whenever possible.

The central question of Popper (1959/2002), at least s *the logic of scientific discovery* (LoSD), was how we can at the same time admit that all our knowledge is fallible and still rationally justify a belief in the possibility of a growth of knowledge. Popper began his scientific career at a point in time when some of the pillars of physics – the showcase project of modernity – had just been scattered by the “Einsteinian revolution.” Hence, one could no longer ignore the possibility that even our most highly valued intellectual tenets, such as Newtonian mechanics, could possibly turn out to be wrong and be replaced by better theories at any point in time. Still, to Popper, it would be just silly to insist that there is no progress, when at the same time, science, technology, and society had just begun to evolve at an unprecedented pace.

Maybe his most important insight was that the concept of truth in an absolute sense is not needed to believe in progress: if a new theory explains everything that an old theory could explain (for example, but not only, in the sense of making correct predictions) and explains additional phenomena that the old theory could not explain – that is progress, simple as that. Science should accordingly be done in a way that facilitates exactly this kind of progress: scientists should make bold predictions that can easily be shown to not correspond with certain observations (falsification), so that one can as easily as possible identify ways to improve upon them (Popper, 1970). The belief in an absolute truth can hence even easily hinder progress, since one cannot improve upon a supposedly absolute truth.

### Critical Rationalism and Modernism/Positivism

Critical rationalism was a response to positivist/inductivist approaches, such as the logical empiricism of the “Vienna circle” that aimed at defining verification criteria, that is, procedures that allow scientists do discern true from false statements. Ideally, scientific knowledge should only (or predominantly) be based upon verified elementary statements (e.g., Reichenbach, 1938; Carnap, 1967/1928). However, as the famous US-American A.J. Ayers logical positivist famously pointed out in a TV-interview with British philosopher Bryan Magee in 1978, logical positivism failed and finally fell into disfavor with epistemologists, because no viable verification procedure could ever be identified (the full interview can be found at PhilosophyOverdose, undated).

Popper also argued against the idea that the social sciences should just “copy” the methods of the natural sciences, such as experiments (Popper, 1976/1969, p. 90). Just like the concept of an absolute truth can hinder progress, methods that appear to guarantee true knowledge can also forestall progress, for example, if methods that are meant to discover discrepancies between expectations and observations are used to “prove” theories. Popper calls this uncritical copying of research methods from the natural sciences “scientism” (ibid.) and “misguided naturalism” (p. 91).

The way in which methods such as experiments are used in psychology to create evidence in favor of theories was maybe most sharply criticized from a falsificationist perspective by another critical rationalist, Imre Lakatos:

After reading Meehl (1967) and Lykken (1968) one wonders whether the function of statistical techniques in the social sciences is not primarily to provide a machinery for producing phoney corroborations and thereby a semblance of 'scientific progress' where, in fact, there is nothing but an increase in pseudo-intellectual garbage (Lakatos, 1978, p. 88).

What social scientists should attempt to copy instead from the “older” natural sciences is the critical approach that can be found often, but not always, among leading physicists and chemists: the possibility of a growth of knowledge critically depends on the willingness of the protagonists within scientific discourse to expose their ideas to criticism and to admit it when discrepancies between their expectations and observations emerge (falsification). They also have to be willing to change their beliefs if someone can offer a better explanation (see above) for the phenomena to be explained. In the section on “progress” below, we will discuss in more concrete terms different interpretations of the implications of a critical rationalist epistemology for the social sciences.

Two aspects which are related to our previous discussion of postmodernism should be noted here: first, Popper readily admits that several mutually incompatible accounts of an event can exist; this seems to me to correspond in many aspects to concept of Lyotard (1979/1984) of a narrative. Second, proponents of different narratives can and should still attempt to exchange views and to – whenever this is possible – reach a consensual position, just like speakers of different languages can at least try to reach a common understanding (Popper, 1978). They make the attempt although there is no guarantee that they will come to a common understanding and although the understanding they reach will certainly likely be less than perfect. Hence, apart from a critical stance, scientific progress also depends critically on the willingness to communicate in a consensus-oriented way.

In the following paragraphs, I will try to summarize and to directly compare the postmodern (mainly sensu Gergen, 1990) and the critical rationalist stance toward three pivotal epistemological concepts: notions of truth, epistemic progress, and the role and function of methods in scientific inquiry.

## Truth

Throughout all of Popper’s works, truth in an absolute or metaphysical sense must be discerned from individual cases where assumptions about the world (theories or hypotheses) apparently correspond to observations. The absolute truth status of scientific hypotheses or theories can never be clarified once and for all. This is because, for example, explanatory hypotheses and theories both refer to an infinite class of phenomena (e.g., every X under condition Y). Hence, even if all observations had so far corresponded to our theories, one can never be sure that all future observations will do so as well (this problem is sometimes called the Humean problem of induction, e.g., Popper, 1959/2002, p. 5).

Additionally, to Popper, there are no theory-free observations: all “facts” that we derive from our senses should be understood as answers to questions that we formulated beforehand or as tentative solutions to problems that we have tried to solve. Hence, all insights from empirical research are necessarily preliminary:

Knowledge does not start from perceptions or observations or the collection of data or facts, but it starts, rather, from problems. One might say: No knowledge without problems; but also, no problems without knowledge. But this means that knowledge starts from the tension between knowledge and ignorance (Popper, 1976/1969, p. 88).

On a side note, it should be noted here that also a falsification of a theory is itself fallible: we could discover at any point, for example, that we ignored some boundary conditions or auxiliary hypotheses which led us to falsely believe that our theory was false.

Popper was also fully aware that the questions we ask or the problems we attempt to solve are culturally bounded: “*Ninth thesis*: A so-called scientific subject is merely a conglomerate of problems and attempted solutions, demarcated in an artificial way. What really exists are problems and solutions and scientific traditions (Popper, 1976/1969, p. 92).”

However, Popper also frequently warned of the dangers of the “malaise of existentialism” (Popper, 1976/1969, p. 104) that could result from an erroneous interpretation of the insight that all our knowledge is fallible: the fact that we cannot know anything for certain and that all insights are to some degree culturally bounded does not allow for the conclusion that one error is just as bad or good as another and that researchers cannot at least attempt to find increasingly better solutions for problems.

Immediately after his “ninth thesis” (see above), Popper tells the story of an interdisciplinary meeting on the future of humanism that he once attended, in which a cultural anthropologist took part as well. In the following paragraph, Popper mocks (in the voice of the anthropologist) the anthropologist’s relativist stance in being unwilling to discuss the arguments that the participants brought forward with regard to the topic of the meeting:

While arguments or reasons make an impression on you, as participants in a discussion, what interests us is the fact that through such means you can mutually impress and influence each other; and also of course the symptoms of this influence. We are concerned with concepts such as emphasis, hesitation, intervention, and concession. We are actually not concerned with the factual content of the discussion but only with the role which the various participants are playing: with the dramatic interplay as such. As to the so-called arguments, they are of course only one aspect of verbal behaviour and not more important than the other aspects (Popper, 1976/1969, p. 94; emphasis as in the original).

It is important to note that what Popper criticizes first and foremost is what he views as the cultural anthropologist’s arrogance in assuming that his understanding of the situation is more objective than the other participants’ viewpoints. He also criticizes the anthropologist’s claim to be able to discern safely between “objective observations” of “verbalizations” and other forms of behavior and objectively invalid pseudo-arguments of the participants that only serve some obscure political purpose. Popper certainly dislikes the unwillingness of the anthropologists to at least attempt to solve the problem at hand and the anthropologist’s cynical ridiculing of any attempt to make progress and to reach a mutual understanding.

To my understanding, Popper does not criticize the *questions* that postmodernists ask of other scientists (and social scientists in particular), such as the question about societal power structures that are at play in academic discourse as well. However, Popper does dislike the fact that postmodernists often (at least, apparently, according to his experience) seem to think that they have an *objective* or otherwise privileged answer to these questions. If a postmodern criticism of a debate such as the one outlined above could and would be formulated as “testable hypotheses” in the sense of debatable and criticizable statements, such criticism could constitute an important element within a critical rationalist attempt to gain an increasingly refined understanding of societal phenomena.

In the last paragraphs, I introduced a term that may be a bit more difficult to understand than Popper’s concept of truth: Popper’s concept of *objectivity*, which played a pivotal role in his later scientific work from the 1960s onward (see, e.g., Popper, 1976/1969). How can there be “objective knowledge” if we cannot have truth? The only answer to this question can be that objectivity is as much of an unreachable regulative ideal as is truth. The opposite of objectivity here would be subjectivity, in the sense that a statement is only comprehensible for me or that an insight about the world makes sense for me, but I cannot communicate it successfully to others (as in Wittgenstein’s concept of a *private language* that cannot be understood by others; Wittgenstein, 1958/1953, $259, p. 92 f.). In this sense, objectivity seems to equal intersubjectivity: a statement can only be objective to the degree that critical, but well-meaning (in the sense that they are not cynical and that they are genuinely interested in finding a solution to the problems that are under discussion) participants will reach a common understanding of the statement and a consensus about the (of course tentative) truth status of the statement at a given point in time within a certain socio-historical context. To Popper, scientists in their discussions should strive for increasing objectivity just as much as they are supposed to strive for the truth. But claims of “absolute” objectivity make just as little sense as do claims for an “absolute” truth. An important tool for achieving increasing objectivity is to Popper the application of formal logical principles.

However, things get complicated through Popper’s frequent attempts to belittle the roles that, for example, societal structures, the socio-historical context or the socio-cultural embeddedness of individual researchers play in academic discourse – and particularly in discourse in the social sciences: “Such minor details as, for instance, the social or ideological habitat of the researcher, tend to be eliminated in the long run; although admittedly they always play a part in the short run” (Popper, 1976/1969, p. 96). It seems that Popper’s horror of a postmodernist relativist skepticism that renders any attempts to solve problems futile makes him sometimes talk as if critical scientists could grasp the objective (not-subjective) aspects of a problem or of proposed solutions in an absolute way. His frequent recourse to formal logical arguments and the possibility of deducting hypotheses from theories can intensify the impression that objectivity can be grasped, whereas truth remains an unreachable ideal. However, such a reading of Popper is, in my opinion, self-contradictory and untenable. In consequence, although Popper frequently expressed dislike toward thinkers such as Foucault (see, e.g., Horgan, 2018), his ideas may have been closer to postmodern thinkers than he was aware of himself (see also Holtz, 2016).

It is interesting to compare Popper’s criticism of relativism with criticism of “objective knowledge” of Gergen (1994) in his response to criticism of Smith (1994) his agenda for a postmodern psychology:

Consider the ideal of objective knowledge. In psychology, as in other sciences, the claim to ‘objective knowledge’ operates as a conversational trump. It disregards or denigrates all hands not dealt in these terms (e.g., evidence, measurement, reliability). Any views not based on scientific tenets—for example, those of sundry religions, political action groups, ethnicities, genders, cultures—can be dismissed as folk beliefs—or more pejoratively, as value-biased, superstitious, or despotic. In terms of its relational implications, ‘science talk’ is thus as totalizing as that of the demagogy that science has sought to replace (Gergen, 1994, p. 413).

But is this the objective knowledge that Popper had in mind? I would think that Gergen is criticizing here the positivist/modernist psychologists’ claim to have access to *actual* objective knowledge, and not so much the use of objectivity as a guiding ideal. Popper would probably agree that claims to objective knowledge are problematic and can indeed be easily abused to justify discrimination and other forms of power games. Popper (1945) criticized exactly this misuse of claims to objective knowledge, for example, in his “open society.” However, it must be noted that whereas Gergen does not want to draw a boundary between science and other societal institutions such as religion, Popper, particularly in his early works, attempted to differentiate between science and non-science on the basis of the falsifiability = criticizability of its tenets (e.g., Popper, 1959/2002, p. 10 ff.). We will come back to this question in the paragraphs on the conceptualizations of progress and the role of empirical research methods.

We finally arrive at the question as to whether Popper’s concept of truth as a regulative ideal can be reconciled with Lyotard’s and Gergen’s postmodernist approaches. I would argue that they are indeed compatible (see also Holtz, 2016; Holtz and Odağ, 2018). If we take, for example, statement of Gergen (1990) that instead of researchers who are just “objectifying the taken-for-granted assumptions of the culture” (p. 33), we need scholars who are “willing to be audacious, to break the barriers of common sense by offering new forms of theory, of interpretation, of intelligibility” (ibid.), this could very well also be a sentence from one of Popper’s later works, as long as these audacious scholars also display humility and the willingness to expose their new theories to criticism.

The same is true for demand of Gergen (1990) that psychologists should focus more on societal problems and on creating a better world: “Required, then, is a form of professional investment in which the scholar attempts to de-objectify the existing realities, to demonstrate their social and historical embeddedness, and to explore their implications for social life.” To my understanding, this statement resembles quite closely Popper’s proposal to improve societies by means of small-scale societal experiments in his “open society” (Popper, 1945).

A non-naive reading of Popper’s use of terms such as truth and objectivity as guiding ideals (be it the understanding Popper intended or not) is indeed compatible with a not completely “radical” postmodernist or constructivism (see also Gadenne, 2008). I mean this in the sense that it is not regarded as outright impossible to reach some form of a common understanding and consensus among well-meaning participants in discourse. Of course, such a consensus is fallible, and it occurs against a certain socio-historical background.

In constructivism, intersubjectivity is needed as well as means of reaching a consensual understanding of social constructions. Hence, attempts at mutual understanding are necessarily at the core of any postmodern research agenda. However, critical rationalism would ask the participants in a discourse to go beyond mutual understanding in that they are also asked to attempt not only to understand each others’ constructions of the world, but also to reach a common understanding with regard to, for example, problems, that are to be solved and the assessment of proposed solutions to these problems. Postmodernism sensu Gergen, in contrast, seems to merely aim at acknowledging and giving voice to different social constructions and world views; critical rationalism also endorses diversity, but attempts should be made at reaching some form of common sense whenever that is possible. Here, postmodernists will most likely be afraid that in the attempt to find common ground and common sense privileged groups will be likely to normatively enforce their world views upon less privileged groups. To the critical rationalist, this is a valid concern, but giving up attempts at reaching a mutual understanding in the sense of consensually negated assessments of problems and proposed solutions would mean to give up any chance for societal or epistemological progress, and this is not an option for the critical rationalist.

## Progress

Progress in the form of replacing theories with better theories is a central concept in critical rationalist thinking. Although to Popper all knowledge is preliminary, he would maintain that one can hardly deny that there has been some form of progress in science that mirrors the increasing complexity of life forms caused by (at least at the level of individuals) seemingly chaotic attempts at propagating genes (see, e.g., Popper, 1971/1961). Deutsch (2011) proposed a maybe even more radical evolutionary epistemology than Popper’s in describing the acquisition of knowledge as an epiphenomenon of life’s evolution: just like life forms evolved from attempts to solve problems such as survival and from the fact that successful adaptations to environments manifested themselves in transmittable DNA structures, our knowledge is the result of millennia of more or less successful attempts at problem solving and attempts to codify the outcomes of these trials in cultural products, such as human language and among others, the cultural tradition called science:

Both in science and in biological evolution, evolutionary success depends on the creation and survival of objective knowledge, which in biology is called adaptation. That is, the ability of a theory or gene to survive in a niche is not a haphazard function of its structure but depends on whether enough true and useful information about the niche is implicitly or explicitly encoded there (Deutsch, 1998, p. 48).

Of course, knowledge is not accumulated in a monotonous way, in the sense that true statements about the world are kept whereas wrong statements are dismissed (for a criticism of this positivistic notion of a cumulative growth of knowledge see Kuhn, 1962, p. 169 ff.). There can always be the kinds of temporary setbacks Kuhn (1962) described in his “structure” (e.g., p. 111 ff.). Totalitarian societal structures that prohibit asking critical questions and trying out new solutions for problems can even forestall any progress whatsoever for some time. However, according to Deutsch (2011, p. 64 ff.), we can observe since the enlightenment an increased speed in the acquisition of knowledge (in the sense of a cultural product) that goes along with societal and political developments, such as increasing freedom and democracy in Western societies. Deutsch (2011) frequently dismisses calls for a change toward more “sustainability” as attempts to restore the anti-progressive totalitarian order of earlier and darker epochs (e.g., p. 434 f.). Deutsch does not deny the existence of problems such as global warming and environmental pollution (e.g., Deutsch, 2011, p. 440 f.), but to him going backward does not constitute a viable attempt at solving these problems. The critical rationalist idea of progress critically relies on a certain degree of optimism, in the sense of belief that at least some of the problems we face can indeed be solved.

Is this now the “grand narrative of progress” (Gergen, 1990, p. 30) to which we cannot return anymore according to Lyotard (1979/1984, p. 60), and which Gergen attempts to “demystify” (Gergen, 1990, p. 33)? What Gergen seems to dislike most about the Western grand narrative is that it silences other voices such as, for example, the voice of less privileged members of a society and the voice of non-Western societies altogether. To some degree, critical rationalists would agree that no voice must be silenced as long as it does not itself demand the silencing of other voices in a totalitarian sense (see Popper, 1945). However, Popper and Deutsch would certainly argue that there are rational, and to some degree objective, reasons to prefer (of course, in a still culturally bounded and fallible way) a Western democratic society with all its deficiencies over, for example, a totalitarian theocracy that is ruled by brutish religious zealots. I would think that postmodernists such as Gergen do not have an easy answer to this argument, since the improvement of life-worlds is one of the central elements of Gergen’s outline of a postmodern psychology:

In the postmodern vein, we find that all languages—even that of the research psychologist—can enter the culture and be used by people to justify, separate, control, and castigate. In effect, for the psychologist there is no escaping matters of moral and political consequence. This being the case, not only is it irresponsible to avoid deliberations on the good, but psychologists should be encouraged to add their voices to the culture's dialogues of “ought.” In many cases this may mean political advocacy—championing causes that one believes good for the culture; in others it may mean culture critique—condemning movements or policies that seem inimical to human welfare (Gergen, 1994, p. 414/415).

Postmodernists criticize the somewhat authoritarian (see also Feyerabend and Oberheim, 2011) modernist narrative of a cumulative growth of knowledge (see above), but critical rationalists do so as well. It should also be noted that neither Lyotard nor Gergen rule out the possibility of some forms of progress, such as technological innovation (e.g., Gergen, 1990, p. 31). However, to Gergen, the main contribution of psychology to society is not so much technology, for example, in the form of new and innovative ways of measuring psychological properties and approaches to the treatment of psychiatric diseases. Instead, the main function of psychology, at least in a modern Western democracy, could be to explore different forms of seeing the world and of being in the world: psychology can bring into the open voices that are the most often marginalized within the mainstream societal discourse. However, I do not think that a critical rationalist would object to this endeavor, as long as it does not lead to the cynical refusal to believe in the possibility of any form of consensus and progress. It is of course also understandable that a physicist such as Deutsch focuses in his epistemology first and foremost on what Gergen calls technological innovation and not so much on social and societal issues.

When it comes to societal progress or attempts at creating a better world in general, idea of small-scale societal experiments of Popper (1945) seems to be not so much different from Gergen’s concept of generative theories (e.g., Gergen, 1978): both concepts have in common that trying out new ways of living and of organizing social life is crucial for societal progress. It is not the case that scientists first create “objective” or “true” knowledge which is then eventually applied, for example, by politicians; first, new solutions to existing problems must be found and tried out. One difference is that critical rationalists such as Popper or Deutsch emphasize more strongly that the resulting ideas from concepts such as generative theory or small-scale societal experiments have to be evaluated critically:

This growth, this self-transcendence, has a rational side and a non-rational side. The creation of new ideas, of new theories, is partly non-rational. It is a matter of what is called ‘intuition’ or ‘imagination’. But intuition is fallible, as is everything human. Intuition must be controlled through rational criticism, which is the most important product of human language. This control through criticism is the rational aspect of the growth of knowledge and of our personal growth. It is one of the three most important things that make us human. The other two are compassion, and the consciousness of our fallibility (Popper, 1978, p. 167).

However, the idea that a critical evaluation of the outcomes of generative theories is needed can be found in Gergen’s writings as well: “As assumptions are sustained or rejected, social life may be altered in ways that may be judged ‘good’ or ‘bad’ from some standpoint” (Gergen, 1978, p. 1356). Again, the two approaches may differ from each other more in terms of prioritization than in terms of substance.

To my understanding, postmodernists and critical rationalists have in common that they first and foremost argue against different forms of totalitarianism and that they both encourage the expression of deviating opinions and different voices. However, critical rationalists seem to abhor primarily the specter of a cynical relativism that renders futile any attempts at mutual understanding and at facilitating a change for the better, postmodernists are more concerned about a dogmatic positivistic scientific culture that considers itself superior and to some degree infallible and that has a tendency to silence critics and alternative approaches. It seems to be that both approaches share similar concerns, but their protagonists may have had personal experiences with different forms of dogmatism, which may have led to different sets of fears and concerns.

## Methods

To Gergen (1990), a postmodernist turn would also have methodological implications: as Gergen (2001) discussed elsewhere, laboratory experiments can most often be regarded as “degraded data” and “myopic” (p. 810) from the perspective of a postmodern psychologist who is interested in exploring the depth and richness of culturally bounded discourse patterns and behavioral repertoires. Qualitative methods of inquiry may often be better suited to explore different constructions of reality and the effects of, for example, certain psychological theories or viewpoints on the emergence of corresponding life-worlds. However, Gergen also mentions the potential of “classical” social psychological laboratory experiments (e.g., Asch, 1956; Milgram, 1974) to incite “public discussion on issues of political and societal significance” (p. 808).

Overall, postmodernist researchers seem to be free in choosing any research method they like as long as they are aware that all scientific inquiries are in the end acts of communication within a culturally bound tradition and a system of meaning making. Hence, no claims for an objective or absolute truth can be deduced from any research method. What research methods can do for the postmodernist is that they can aid her in understanding – from her culturally bounded position – the plurality of culturally embedded psychological realities and the ways in which such realities can change under certain conditions.

To Popper (1976/1969), it would of course be foolish to think of certain research methods as pathways to the truth or to exclude certain research methods on ideological grounds. To the critical rationalist, the role and function of empirical research methods are to allow for criticism of theories in the form of giving them a chance to fail. For example, Popper’s whole philosophy is not based on empirical studies, but on thought experiments as well as on formal logic and other rational arguments. Every scientific discipline will need a range of different methods to expose their respective theories to criticism. Hence, one can very well imagine that different research methods are more or less useful in different areas of the so-called natural and social sciences, which can be no more than loosely defined traditions to the critical rationalist.

However, truth and objectivity should be the guiding ideals in the critical rationalists’ choice of methods. What does this mean and what would be the implications for the social scientist? Research methods should help us to overcome “psychologism” (e.g., Popper, 1959/2002, p. 7), that is the idea that insights that appear to be true to the beholder cannot be shared with others in a form that allows for mutual understanding and criticism. It should be noted that this kind of objectivity in the sense of intersubjectivity is also one of the main goals of literally all approaches within the wide field of qualitative research methods (see also Holtz and Odağ, 2018).

Popper himself certainly favored different kinds of research methods in the social sciences. In the “logic of the social sciences,” Popper (1976/1969, p. 103) briefly recommends the kind of “situational analysis” that is used in economics as a possible approach for the social sciences: here, the researcher assumes that human beings behave (more or less) rationally and tries to identify the situational factors under which a certain type of behavior would be rational. It seems to follow that also systematic deviations from the assumption of rationality, for example, in the form of “biases” (e.g., Tversky and Kahneman, 1974) can be analyzed, and the results of these analyses can be used to improve situational analyses.

From a postmodern perspective, this approach to social scientific research can be critziced, for example, because it may tend to ignore the socio-cultural boundedness of social practices and the constructedness of rationality itself. Maybe a critical rationalist would respond that as long as such criticism is presented in a way that it yields debatable or empirically testable assumptions, it can perfectly well be reconciled with a critical rationalist approach to research. Personally, I think that Popper’s apparent predilection for a more homo oeconomicus orientied approach in the social sciences is certainly not the only way in which critical rationalist social scientific research can play out. If we look, for example, at ideas of Pettigrew (1991) on a critical rationalist social psychology, he is envisioning a stronger unity between the different branches within social psychology such as experimental social psychology and a more qualitatively oriented “symbolic interactionist” (p. 13) approach. He also suggests trying to build bridges to more humanities-based approaches in sociology. Hence, a critical rationalist approach to psychology could also be imagined as a more inclusive enterprise bridging the existing gaps between qualitative and quantitative approaches above and beyond the mere “mixing” of methods (cf. Holtz and Odağ, 2018). Just like in the previous paragraphs on truth and progress, Popper’s (or any other philosopher’s) personal preferences do not count much in view of the question how their philosophical approaches can and should be interpreted.

Taken together, postmodernists as well as critical rationalists take a pragmatic stance when it comes to research methods. Postmodernists tend to prefer research methods that allow for the reconstruction of different forms of discourse, whereas most critical rationalists may value approaches that allow for increasingly objective arguments away from intersubjectivity. Still, there do not seem to be any irreconcilable differences here.

## Implications for the Field of Psychology

It is fairly easy to find a common enemy of postmodernists and critical rationalists: modernist psychologists who insist that only experiments (or other research methods that are supposedly borrowed from the hard natural sciences) guarantee true knowledge. By insisting this, they immunize their theories against criticism since any objection that does not result from experiments can be easily devalued as being unscientific. This is particularly worrying since experiments were almost eclusively used over the last decades in the field of psychology to “prove” or to “sell” theories, and not to subject them to severe tests as means of improving on them (Holtz, 2020). Thus, modernist psychologists unfairly make their worldview privileged, excluding other voices from the perspective of the postmodernists. Such approaches can easily be misused as propaganda tools in socio-political power struggles.

Both postmodernists and critical rationalists would ask scientists to be bold, to try out new ways, and to bring diverging opinions to the front. Science is not about being timid and hiding behind pompous technical language (Billig, 2013) or haughty and complicated research methods. Science is – or should be – an adventure (cf. Willig, 2001), and scientists should have the audacity to ask questions that have never been asked before and to try out new solutions to old problems. No knowledge, no theory, and no empirical research is sacrosanct; everything can and must be questioned at any time. I do not see much of a difference between both approaches here.

Both approaches also encourage social scientists to think of themselves as parts of the world and not as passive and objective observers. Popper writes in the “logic of the social sciences”:

Serious practical problems, such as the problems of poverty, of illiteracy, of political suppression or of uncertainty concerning legal rights were important starting points for research in the social sciences. Yet these practical problems led to speculation, to theorizing and thus to theoretical problems. In all cases, without exception, it is the character and the quality of the problem—and also of course the boldness and originality of the suggested solution—which determine the value, or the lack of value, of a scientific achievement (Popper, 1976/1969, p. 89).

However, to Popper, progress in science is always progress in the forms of the development of increasingly “better” theories. The solving of societal problems can probably only be a byproduct of social scientific research. Gergen (1990) goes to great length in his texts to avoid any notion of this kind of progress: all knowledge that might result from any scientific activity can only be understood within its own cultural tradition and cannot claim superiority over other forms of knowledge that resulted from other cultural traditions. However, in view of Gergen’s optimism that politically and societally a change for the better can be achieved, I find it difficult to believe that he really rules out such positive developments for the realm of scientific knowledge. In the following statement, for example, Gergen is discussing the merits of the newly emerging field of theoretical psychology:

The point of criticism should not be that of terminating traditions or practices but of helping them to evolve in ways that more fully integrate the voices of the discipline and of its constituents and contribute to the intellectual resources of the world (Gergen, 2001, p. 809).

To me, it is difficult to think of a successful contribution to the intellectual resources of the world without employing some concept of better and worse contributions. However, Gergen would probably maintain that this concept itself is culturally and temporally bounded. Popper and Deutsch would agree that no claim to knowledge can be “objective,” but they would probably use formal logic and other “rational” arguments to support their belief in the possibility of getting closer to the truth by means of constant trial and error. Here, it would be up to the postmodernists to answer the question whether their relativism with regard to a growth of knowledge is primarily meant to be an attack against naïve positivist modernist psychologists or whether they really believe “in the depth of their hearts” that there cannot be progress, or that objectivity and truth are not ideals that are worth striving for.

On the other hand, critical rationalists such as Popper and Deutsch will have to justify their optimism in believing that striving for truth and objectivity will finally lead to primarily positive consequences. At the same time, critical rationalists have to be aware of the dangers that even well-meaning attempts to contribute to the creation of a better world can turn out to be disastrous and that attempts at reaching a mutual understanding among different voices can unwillingly lead to the establishment of cultural hegemony. Although Deutsch, in particular, sometimes speaks as if the superiority of post-enlightenment Western culture was an “objective fact” (see the quote in the section Introduction), the concepts of fallibility and error correction do of course apply here as well.

## Conclusion

In the end, there are many commonalities between critical rationalist and postmodern approaches: a dislike for modernist arrogance, an emphasis on boldness and innovation, and a struggle against totalitarian attempts to oppress voices in a discourse. Both approaches value attempts by social scientists to address real world problems and to challenge dogmas and established world views. Critical rationalists and postmodernists are both aware that all our knowledge is temporally and culturally bounded, insofar as we can only perceive the world from a certain discursive formation (Foucault, 1969/1972) or that we can only ask questions and receive empirical answers to these questions from a certain point in time within given cultural structures.

The differences between critical rationalists and postmodernists boil down to differences in preferences and predilections: whereas postmodernists sometimes challenge arrogant modernist conceptualizations in a cynical way, critical rationalists prefer to propagate optimism with regard to the question as to whether there are at least some problems that we, as scientists, may be able to solve in a tentative and preliminary way. Whereas postmodernists are mostly afraid of dogmatic empiricists that hide their own political agenda behind claims for objective truth, critical rationalists are first and foremost weary of zealous postmodernists who themselves hide *their* political agenda behind their own claims of objectivity. Whereas postmodernists value a plurality of different voices, critical rationalists hope for a consensual resolution of conflicts by means of relying of increasingly objective arguments.

On a personal note, I do not think that any of these differences are beyond reconciliation. Both approaches value open and free discussions above everything else, and this fact alone should provide common ground for attempts at increasing mutual understanding.

## Author Contributions

The author confirms being the sole contributor of this work and has approved it for publication.

### Conflict of Interest

The author declares that the research was conducted in the absence of any commercial or financial relationships that could be construed as a potential conflict of interest.
